# Connectomic analysis of taste circuits in *Drosophila*

**DOI:** 10.1038/s41598-025-89088-9

**Published:** 2025-02-12

**Authors:** Sydney R. Walker, Marco Peña-Garcia, Anita V. Devineni

**Affiliations:** 1 Department of Biology, Emory University, Atlanta, GA 30322 USA; 2 Neuroscience Graduate Program, Emory University, Atlanta, GA 30322 USA

**Keywords:** *Drosophila*, Taste, Neural circuit, Connectome, Gustatory system, Neural circuits, Sensory processing

## Abstract

Our sense of taste is critical for regulating food consumption. The fruit fly *Drosophila* represents a highly tractable model to investigate mechanisms of taste processing, but taste circuits beyond sensory neurons are largely unidentified. Here, we use a whole-brain connectome to investigate the organization of *Drosophila* taste circuits. We trace pathways from four populations of sensory neurons that detect different taste modalities and project to the subesophageal zone (SEZ), the primary taste region of the fly brain. We find that second-order taste neurons are primarily located within the SEZ and largely segregated by taste modality, whereas third-order neurons have more projections outside the SEZ and more overlap between modalities. Taste projections out of the SEZ innervate regions implicated in feeding, olfactory processing, and learning. We analyze interconnections within and between taste pathways, characterize modality-dependent differences in taste neuron properties, identify other types of inputs onto taste pathways, and use computational simulations to relate neuronal connectivity to predicted activity. These studies provide insight into the architecture of *Drosophila* taste circuits.

## Introduction

Our sense of taste is critical in helping us determine what foods to eat. Attractive tastes indicate that food contains calories or nutrients, while aversive tastes warn that the food may be toxic or spoiled. Most animals, including humans, recognize five basic tastes: sweet, umami (savory), salty, bitter, and sour. In general, taste sensory cells express receptors for individual taste modalities and thus respond to specific tastes^1^. In mammals, signals from taste sensory cells in the tongue are transmitted to the brainstem, thalamus, and gustatory cortex^2^. Neural recordings have revealed a variety of response types across these areas, including cells that respond to specific tastes (“specialists”), cells that respond broadly to multiple tastes (“generalists”), and cells with varying response dynamics^3^. Despite this progress, it is not clear how these different types of neuronal responses arise from the connectivity of the circuit or how specific response types contribute to behavior. Addressing these questions requires a system where we can examine the connectivity, response properties, and behavioral contribution of individual cell types, thus determining how these properties are related.

The fruit fly *Drosophila melanogaster* represents such a system. Recent studies have generated a brain-wide map of neuronal connectivity^4^ and classified the ~ 140,000 brain neurons into thousands of cell types^5^, each typically consisting of just one or a few cells that can be genetically targeted using collections of transgenic driver lines^6–11^. Taste cells in *Drosophila* are distributed throughout multiple organs, including the proboscis (the feeding organ), legs, and wings^12^. The major taste organ that regulates feeding is the labellum, located at the distal end of the proboscis. Several classes of gustatory receptor neurons (GRNs) have been identified in the labellum, including sugar-sensing, bitter-sensing, water-sensing, high salt-sensing, and IR94e-expressing neurons^12–14^. Sugar GRNs promote feeding, whereas bitter and high salt GRNs suppress feeding^13,15^. Water-sensing GRNs detect solutions of low osmolarity and promote water consumption^16^. IR94e GRNs respond to low salt concentrations and amino acids, and these GRNs suppress feeding while promoting egg-laying^13,17,18^.

In contrast to our understanding of taste coding at the periphery, downstream taste circuits in the *Drosophila* brain have remained largely unknown. Labellar GRNs project axons into the subesophageal zone (SEZ) of the brain^19,20^, but their postsynaptic partners have remained a mystery for decades. The development of trans-Tango, a trans-synaptic tracing method, enabled the visualization of neurons that receive synaptic input from GRNs, termed second-order taste neurons^21^. Tracing the postsynaptic partners of sugar or bitter GRNs revealed large populations of second-order neurons with extensive projections in the SEZ and a few major projections to higher brain regions^21–23^. Second-order sugar and bitter neuron populations are anatomically similar and appear to include some overlapping neurons^22^. However, the large number of neurons labeled by trans-Tango made it difficult to determine the extent of overlap and, more generally, to identify individual types of second-order neurons.

The recent release of the first *Drosophila* whole-brain connectome^4^ makes it possible to identify the inputs and outputs of every neuron, enabling us to trace the flow of taste information through the brain. Two initial studies (using connectome data prior to public release) identified labellar GRNs in the connectome dataset^24^ and traced a circuit connecting sugar GRNs to a motor neuron that drives proboscis extension, which represents the initiation of feeding^25^. The sugar proboscis extension circuit comprises a five-layered circuit located entirely within the SEZ^25^. An additional study simulated whole-brain activity using a connectome-based model and predicted which neurons are activated by each taste modality, finding substantial overlap between the neurons activated by sugar and water GRNs but not other modalities^17^.

Several fundamental questions regarding the architecture of *Drosophila* taste circuits remain unanswered. How much taste processing occurs within the SEZ, and which higher brain regions are involved? How much overlap exists between circuits for processing different taste modalities, and does overlap increase or decrease at subsequent layers of processing? To what extent do taste circuits consist of feedforward excitation, feedforward inhibition, or lateral and feedback connections? What other types of inputs are integrated by taste circuits?

In this study, we addressed these questions by analyzing the whole-brain connectome released by the FlyWire consortium^4,5^, which contains neurotransmitter predictions for each neuron^26^. We first traced the postsynaptic partners of GRNs to identify second-order taste neurons. We found that second-order neurons are primarily located within the SEZ, are largely segregated by modality, and show modality-dependent differences in the location and sign of their output projections. Third-order taste neurons showed more overlap between modalities and more projections outside the SEZ, primarily innervating brain regions in the superior protocerebrum in modality-specific patterns. Second- and third-order neurons had numerous lateral and feedback connections and integrated various types of non-taste inputs. Finally, simulations of whole-brain activity revealed relationships between connectivity and predicted responses. Together, these studies provide insight into the architecture of circuits for taste processing in the fly brain, laying the groundwork for functional studies.

## Results

### Second-order taste neurons are largely modality-specific

**Fig. 1:**
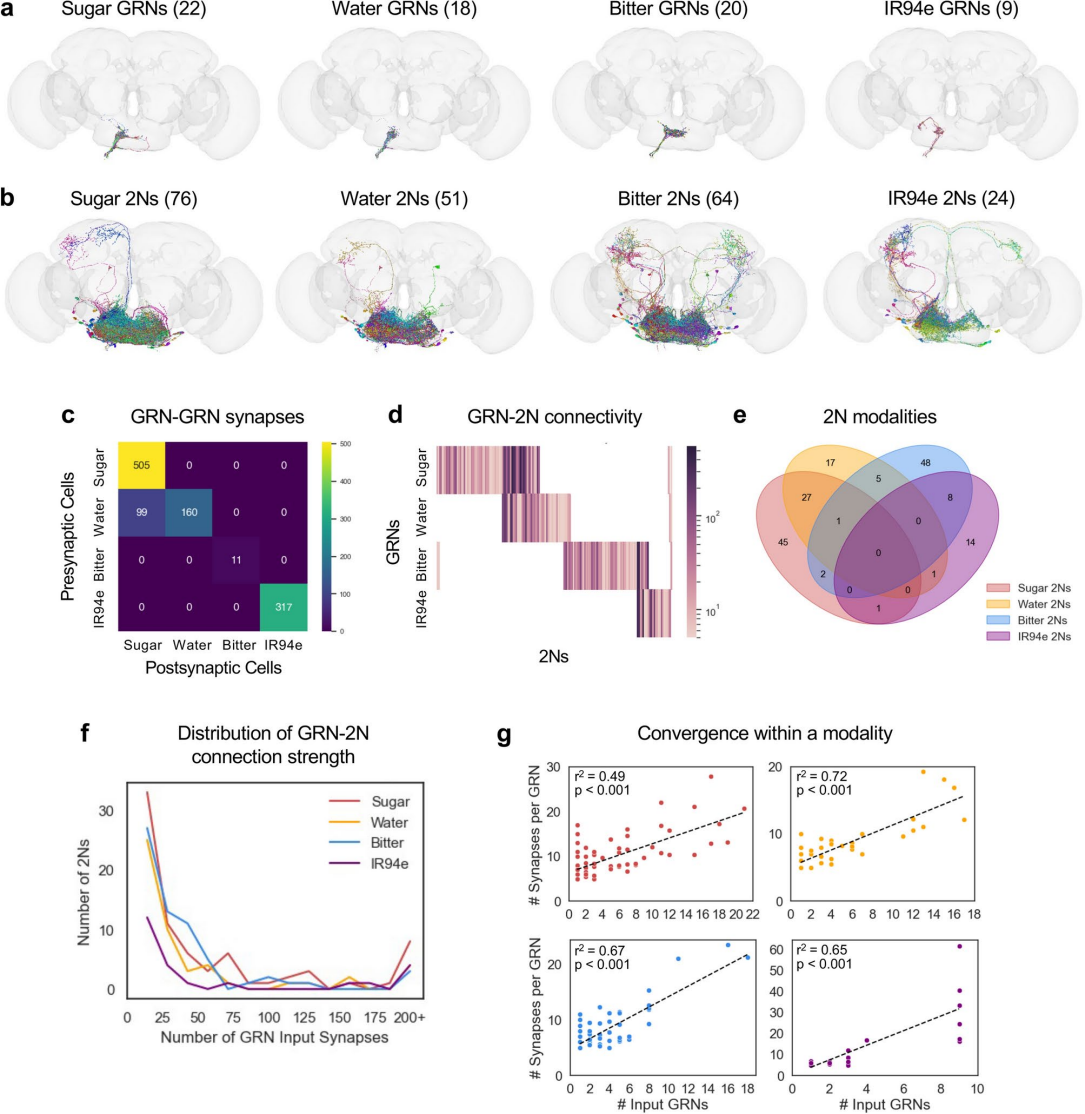
2Ns are largely specific to individual taste modalities. **a**, **b** Images of GRNs (**a**) and 2Ns (**b**) from each of the four taste modalities. Numbers in parentheses denote the number of neurons in each category. Note that neuron images from FlyWire are always left–right inverted. **c** Heatmap showing the number of GRN-GRN synapses for each pair of modalities. **d** Heatmap showing GRN-2N connectivity for each individual 2N. The colorbar represents the total number of input synapses from each GRN type. **e** Venn diagram showing overlap between 2Ns of different modalities. **f** Distribution of GRN-2N connection strength (number of total GRN input synapses to each 2N) for each modality. **g** Correlation between the number of input GRNs for each 2N and the average strength (number of synapses) of each input connection. Ordinary least squares regression was used to obtain r^2^ values and *p*-values.

We started by tracing the postsynaptic partners of labellar GRNs, focusing on the four major GRN classes that have been previously annotated in the connectome: sugar-sensing, bitter-sensing, water-sensing, and IR94e-expressing neurons. We identified GRNs in the connectome based on GRN lists from recent studies^17,18^, neuron annotations in FlyWire, and manual inspection of each neuron’s morphology (see Methods). We limited our analyses to GRNs on the left side of the labellum, which project to the left hemisphere of the brain, because these neurons have been more thoroughly annotated and we are more confident of their identity. Our GRN lists include 22 sugar GRNs, 18 water GRNs, 20 bitter GRNs, and 9 IR94e-expressing GRNs (Fig. 1a; note that neuron images from FlyWire are left–right inverted^4,5^). Sugar and water GRNs project to overlapping regions of the SEZ, whereas the projections of bitter and IR94e GRNs are more distinct. GRNs are thought to be excitatory, which aligns with most of the neurotransmitter predictions in FlyWire^26^ and experimental data showing that second-order sugar neurons are activated by sugar^25^.

We used the connectome to identify second-order taste neurons (2Ns), neurons that receive direct input from GRNs. Using a connection threshold of 5 synapses, a threshold suggested by FlyWire in order to exclude false positives^4^, we identified 76 sugar 2Ns, 51 water 2Ns, 64 bitter 2Ns, and 24 IR94e 2Ns (Fig. 1b). These numbers correspond to approximately three times as many 2Ns as GRNs, with 2N/GRN ratios ranging from 2.7 for IR94e to 3.5 for sugar.

We also identified many GRN-GRN connections, which have been described in a previous study^24^. The majority of these connections were between GRNs of the same type, with the exception of water GRNs synapsing onto sugar GRNs (Fig. 1c). When normalized by the number of GRNs, within-type connections were most prominent for IR94e (35 synapses/GRN), followed by sugar GRNs (23 synapses/GRN) and water GRNs (9 synapses/GRN), and they were almost nonexistent for bitter GRNs (< 1 synapse/GRN). Thus, some taste modalities exhibit more GRN crosstalk than others. Note that GRNs were excluded from our lists of 2Ns, as our goal was to trace feedforward taste pathways.

The 2Ns for each taste modality were largely distinct, with the exception of strong overlap between sugar and water 2Ns (28 neurons) and weaker overlap between bitter and IR94e 2Ns (8 neurons) and between bitter and water 2Ns (6 neurons) (Fig. 1d–e). These results suggest an early segregation between taste modalities that are appetitive (sugar and water) or aversive (bitter and IR94e) in the context of feeding, which we will refer to here as “appetitive” or “aversive” modalities even though they may have different roles in distinct behavioral contexts^12,13,15–18^.

Within a modality, the strength of GRN input to each 2N varied widely, with most 2Ns receiving less than 20 total GRN synapses while some 2Ns received over 100 synapses (Fig. 1f). 2Ns received a median of 15–17 total synapses from a median of 2–3 GRN input cells, depending on modality. Interestingly, 2Ns receiving input from more GRNs also tended to receive more synapses per GRN input cell, whereas fewer input GRNs corresponded with weaker connections (Fig. 1g; r^2^ values ranged from 0.49 to 0.72). This correlation would tend to widen the distribution of GRN-2N connection strength, creating a larger separation between 2Ns receiving few versus many GRN inputs. Together, these analyses show that 2Ns are largely segregated by modality—with the notable exception of sugar and water convergence—and vary widely in the strength of the taste input they receive.

### 2Ns show modality-specific crosstalk and feedback

**Fig. 2:**
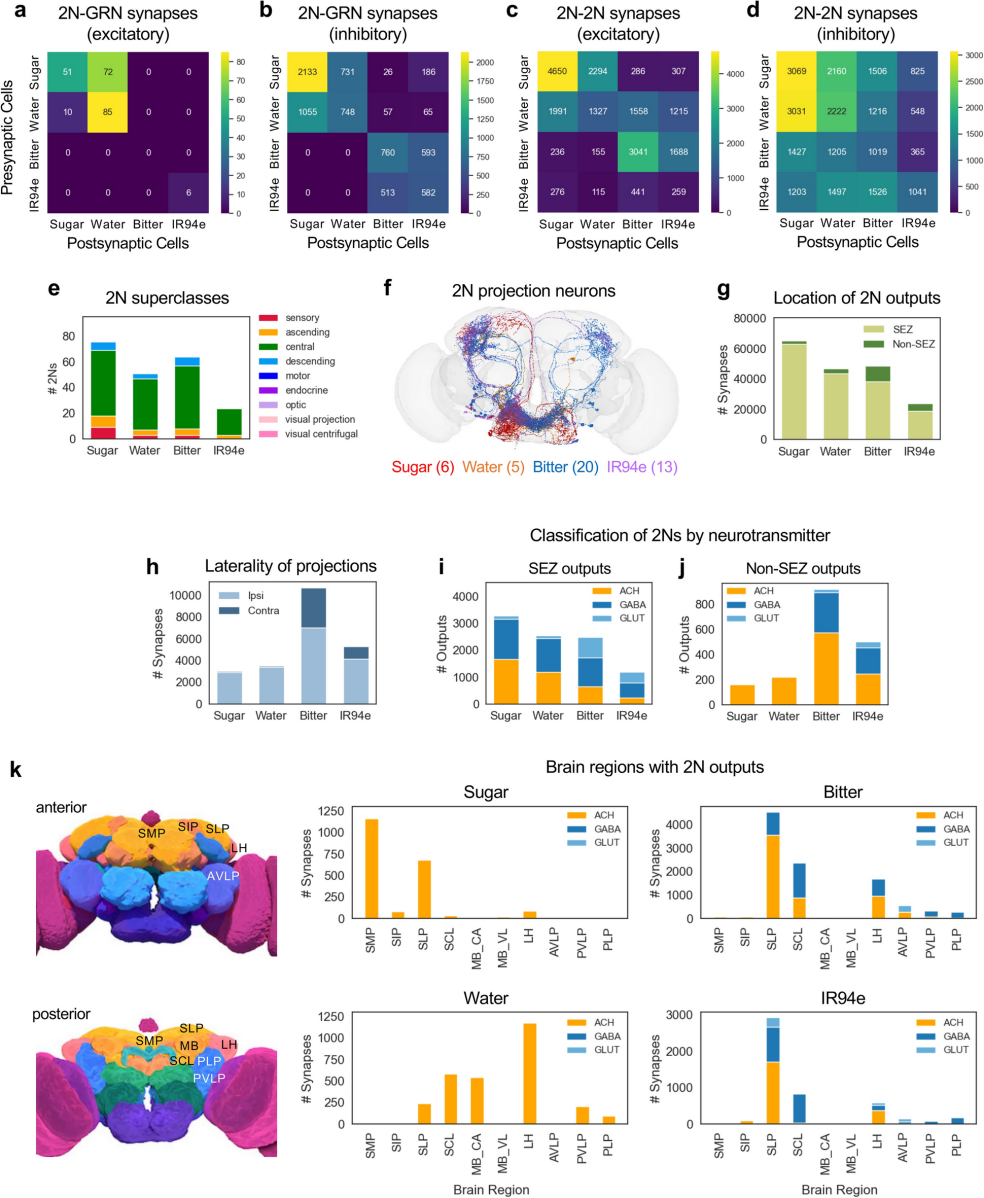
Anatomical and functional properties of 2Ns vary by taste modality. **a**–**d** Heatmaps showing the number of 2N output synapses onto GRNs (**a**–**b**) or other 2Ns (**c**–**d**). Excitatory and inhibitory synapses are shown in separate plots. **e** Number of 2Ns belonging to each of the 9 superclasses. Only the first 4 superclasses are represented in the 2N population. **f** Image showing 2N projection neurons for each modality. Neurons are shown at 50% opacity to visualize neurons at multiple depths and enable intermediate coloring for neurons belonging to more than one modality, although these represented a very small proportion of projection neurons. **g** Number of 2N output synapses that reside within versus outside of the SEZ. All pairwise comparisons of SEZ/non-SEZ proportions between modalities were significantly different except for bitter vs. IR94e (Fisher’s exact test). **h** Number of 2N output synapses outside the SEZ that are located in the ipsilateral versus contralateral hemisphere (relative to the location of GRN projections). All pairwise comparisons of ipsilateral/contralateral proportions between modalities were significantly different except for sugar vs. water (Fisher’s exact test). **i**–**j** Predicted neurotransmitters for 2N local (**i**) or projection (**j**) output connections (ACH, acetylcholine; GLUT, glutamate). All pairwise comparisons of the proportion of excitatory (ACH) versus inhibitory (GABA and GLUT) outputs between modalities were significantly different except for sugar vs. water non-SEZ outputs (Fisher’s exact test). **k** Number of 2N output synapses in brain regions outside of the SEZ, color-coded by neurotransmitter type. The top 6 brain regions for each modality are included, comprising a total of 10 regions that include > 96% of non-SEZ output synapses for each modality. Pictures on the left show the approximate location of each brain region (images are from the FlyWire website). See text and Methods for abbreviations.

To assess whether feedback or lateral connections are prominent at the second layer of the taste circuit, we asked whether 2Ns send outputs back to GRNs or to other 2Ns. Because excitatory and inhibitory connections would have different roles, we classified 2N outputs by their predicted neurotransmitter type^26^ and analyzed excitatory and inhibitory connections separately. In the *Drosophila* central nervous system, the major excitatory neurotransmitter is acetylcholine and the major inhibitory neurotransmitters are GABA and glutamate^27,28^. We found that 2Ns had many feedback connections onto GRNs, with inhibitory connections being much more common (Fig. 2a, b). Excitatory 2N-GRN connections occurred almost exclusively within or between the sugar and water pathways (Fig. 2a). Inhibitory 2N-GRN connections primarily occurred within each modality, between the sugar and water pathways, or between the bitter and IR94e pathways (Fig. 2b). These data reveal that 2N-GRN feedback preferentially occurs between the two appetitive pathways (sugar and water) and between the two aversive pathways (bitter and IR94e), similar to the pattern of GRN-2N convergence (Fig. 1e).

2Ns also had many lateral connections with each other. Excitatory 2N-2N connections commonly occurred within the same modality, particularly for sugar and bitter (Fig. 2c). Cross-modal excitatory connections also occurred between sugar and water 2Ns, from water 2Ns to bitter and IR94e 2Ns, and from bitter 2Ns to IR94e 2Ns (Fig. 2c). In contrast, inhibitory 2N-2N connections were broader and often cross-modal, with substantial crosstalk between every pair of modalities (Fig. 2d). Thus, cross-inhibition between different taste modalities is a prominent motif that occurs early in the taste circuit.

### Properties of 2Ns vary by taste modality

We next examined the anatomical and predicted functional properties of 2Ns. First, we identified the “superclass” of each 2N. The 9 superclasses are defined by the location of a neuron’s inputs and outputs^5^, and all 2Ns belonged to one of 4 superclasses: sensory (projecting from sensory organs to the brain), ascending (projecting from the ventral nerve cord to the brain), central (projecting exclusively within the central brain), or descending (projecting from the brain to the ventral nerve cord) (Fig. 2e). The majority of 2Ns were central neurons (67–88%, depending on modality), but ascending and descending neurons each comprised 8–13% of 2Ns with the exception of the IR94e 2Ns, which did not include any descending neurons (Fig. 2e).

We also identified the “class” of each 2N, which refers to a grouping of neurons that has been characterized in the literature^5^. However, less than half of neurons in the central brain have been assigned to a class^5^, and the majority of 2Ns are unassigned (Table 1). 2Ns with class assignments belonged to one of 4 classes: gustatory (i.e., GRNs, which represent GRNs not annotated in this study because we excluded our annotated GRNs from 2N lists), ascending neurons, descending neurons, or antennal lobe projection neurons (ALPNs), with the latter class mainly including bitter 2Ns (Table 1).

**Table 1:** 2N and 3N classes.

Class	2Ns				3Ns			
	Sugar	Water	Bitter	IR94e	Sugar	Water	Bitter	IR94e
gustatory	9	3	3	0	59	32	56	30
mechanosensory	0	0	0	0	9	0	0	0
AN (ascending)	9	4	5	3	17	6	18	13
DN (descending)	7	4	7	0	89	45	34	13
motor	0	0	0	0	37	7	12	3
pars intercerebralis	0	0	0	0	0	0	3	3
ALIN (antennal lobe input neurons)	0	0	0	0	1	3	0	0
ALPN (antennal lobe projection neurons)	0	1	6	0	2	3	0	0
mAL	0	0	0	0	2	0	8	7
LHCENT (lateral horn centrifugal neurons)	0	0	0	0	0	0	6	2
Kenyon cell	0	0	0	0	0	28	0	0
unknown (not annotated)	51	39	43	21	299	200	259	151
Total	76	51	64	24	515	324	396	222

Table entries list the number of 2Ns or 3Ns of each modality belonging to each class.

Next, we analyzed the extent to which 2Ns process information within versus outside of the SEZ. We classified 2Ns as local neurons, whose outputs reside exclusively within the SEZ, or projection neurons, which have at least some outputs outside of the SEZ. The majority of 2Ns were local neurons, and the number of projection neurons varied by modality: projection neurons comprised only 8–10% of sugar or water 2Ns (5–6 cells) but comprised 31% of bitter 2Ns (20 cells) and 54% of IR94e 2Ns (13 cells) (Fig. 2f). Consistent with these numbers, the majority of 2N output synapses were located within the SEZ, and the proportion of output synapses outside of the SEZ was much higher for bitter and IR94e 2Ns (21%) than water and sugar 2Ns (3–6%) (Fig. 2g). Most 2N output synapses outside of the SEZ were located in the ipsilateral hemisphere of the brain (relative to the location of GRN projections), but bitter and IR94e 2Ns had a much higher proportion of contralateral outputs (34% and 22%, respectively) than sugar and water 2Ns (3%) (Fig. 2h).

We then analyzed the predicted neurotransmitter types of 2N outputs within and outside the SEZ. The ratio of excitatory and inhibitory outputs within the SEZ was roughly equal for sugar and water 2Ns (49–53% inhibitory) but was biased toward inhibition for bitter (73% inhibitory) and IR94e (81% inhibitory) 2Ns (Fig. 2i). Interestingly, 2N outputs outside of the SEZ had a different distribution that was more skewed toward excitation: they were only 2% inhibitory for sugar and water 2Ns and 38% or 51% inhibitory for bitter or IR94e 2Ns, respectively (Fig. 2j). Thus, we observe differences in the predicted sign of 2N outputs that depend on the location and modality of the projections.

Finally, we analyzed the anatomical locations of 2N projections outside the SEZ. Projection 2Ns had output synapses in a variety of brain regions, and the location of outputs varied by modality (Fig. 2k). Bitter and IR94e projection 2Ns, which make up the majority of projection 2Ns, had outputs predominantly in the superior lateral protocerebrum (SLP), superior clamp (SCL), and lateral horn (LH). Sugar projection 2Ns had outputs primarily in the SLP and the superior medial protocerebrum (SMP), and water projection 2Ns had outputs in the LH, SCL, SLP, and mushroom body calyx. Together, these analyses reveal that 2Ns primarily convey taste information within the SEZ but also project to a small number of higher-order brain regions in a modality-specific manner.

### Third-order taste neurons overlap across modalities

**Fig. 3:**
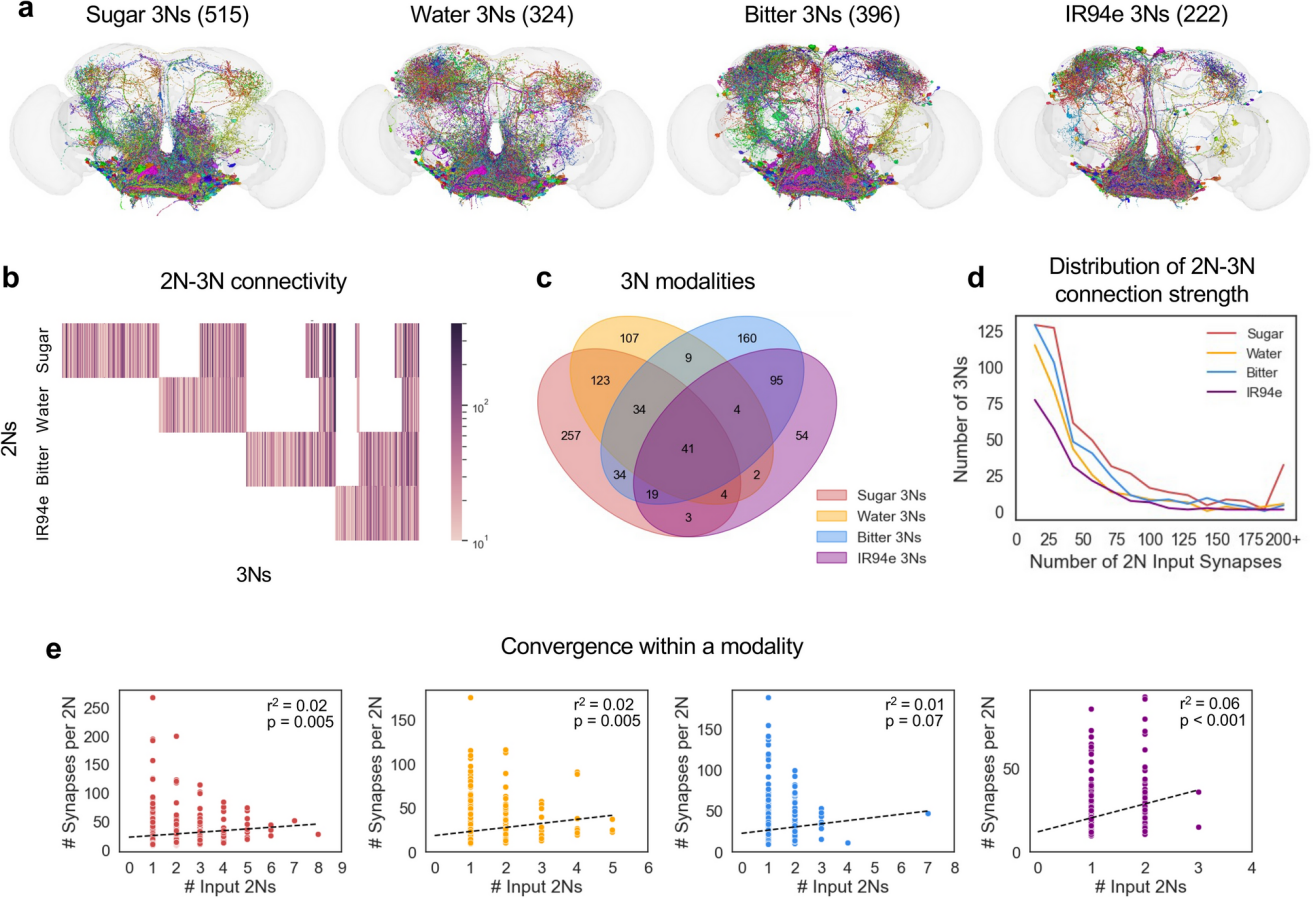
3Ns overlap across taste modalities. **a** Images of 3Ns for each of the four taste modalities. Numbers in parentheses denote the number of 3Ns. **b** Heatmap showing 2N–3N connectivity for each 3N. The colorbar represents the total number of input synapses from each 2N modality. **c** Venn diagram showing overlap between 3Ns of different modalities. **d** Distribution of 2N–3N connection strength (number of total 2N input synapses to each 3N) for each modality. **e** Correlation between the number of input 2Ns for each 3N and the average strength (number of synapses) of each input connection. Ordinary least squares regression was used to obtain r-squared values and *p*-values.

Next, we traced the outputs of 2Ns in order to identify third-order taste neurons (3Ns). Because iterative tracing of connectivity generates larger and larger numbers of neurons at each layer, we set a more stringent threshold for 3N identification: we only traced the partners of 2Ns receiving at least 10 total synapses from GRNs of the same modality, and we similarly set a 10 synapse cutoff for 2N to 3N connections within a modality. Our list of 3Ns excludes GRNs of any type as well as neurons that are 2Ns for the same modality, but a 3N could be a 2N for a different modality. With these criteria, we identified 515 sugar 3Ns, 324 water 3Ns, 396 bitter 3Ns, and 222 IR94e 3Ns (Fig. 3a).

3Ns showed much more overlap between taste modalities than 2Ns, with some overlap between every possible set of modalities (Fig. 3b, c). For example, of the 515 sugar 3Ns, 39% were water 3Ns, 25% were bitter 3Ns, and 13% were IR94e 3Ns. The majority of 3Ns received input from more than one modality (50% of sugar 3Ns, 67% of water 3Ns, 60% of bitter 3Ns, and 76% of IR94e 3Ns).

Within a modality, 3Ns received a median of 22–29 total synapses (depending on modality) from a median of one 2N (Fig. 3d), representing a lower rate of convergence than observed from GRNs to 2Ns (but note that the criteria for 2N-3N connectivity were more stringent). Unlike GRN to 2N synapses, for 2N to 3N synapses there was no clear correlation between the number of input cells and the strength of each input connection (Fig. 3e; r^2^ values ranged from 0.01–0.06).

**Fig. 4:**
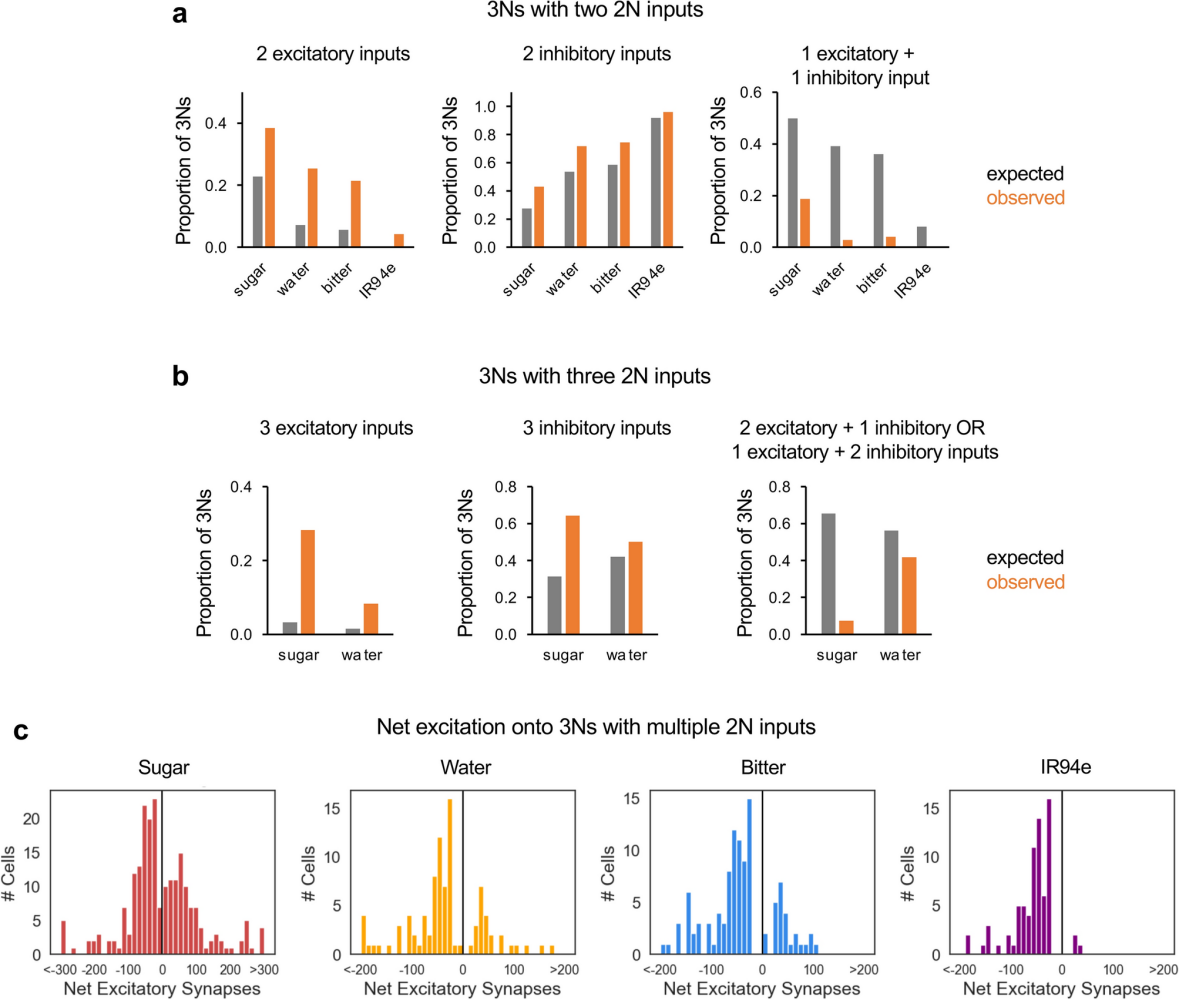
Within a modality, 2N inputs of the same sign tend to converge onto 3Ns. **a** 3Ns receiving exactly two 2N inputs from the same modality were analyzed. The expected proportion of 3Ns receiving two excitatory inputs (left), two inhibitory inputs (middle), or one excitatory and one inhibitory input (right) was compared to actual proportions. For each of the four modalities, the observed proportion of 3Ns in each category differed significantly from expected proportions (*p* < 0.001, chi-squared test; n = 71–112 3Ns depending on modality). **b** 3Ns receiving exactly three 2N inputs from the same modality were analyzed. The expected proportion of 3Ns receiving three excitatory inputs (left), three inhibitory inputs (middle), or a combination of excitatory and inhibitory inputs (right) was compared to actual proportions. We only included modalities for which we could analyze at least 10 3Ns (sugar and water). The observed proportion of 3Ns in each category differed significantly from expected proportions for sugar (*p* < 0.001, chi-squared test; n = 53 3Ns) but not for water, which had a small sample size (n = 12 3Ns). **c** Distribution of net excitation onto each 3N receiving multiple 2N inputs within a modality. Net excitation represents the difference between the number of excitatory and inhibitory 2N input synapses; positive numbers represent net excitation and negative numbers represent net inhibition.

We analyzed whether 2Ns from the same modality that converge onto common 3Ns tend to provide input of the same sign. Convergent input of the same sign would lead to net excitation or inhibition, whereas convergent input of opposite signs would be expected to cancel out. Focusing on 3Ns that receive convergent input from two or three 2Ns of the same modality, which represent 86% of all 3Ns receiving multiple 2N inputs, we found that the frequency of convergent input of the same sign (all excitatory or all inhibitory) was always higher than the frequency expected by chance, especially for excitatory convergence (Fig. 4a, b). We also calculated the net excitation onto each 3N receiving multiple 2N inputs within a modality by quantifying the difference in the total number of excitatory and inhibitory input synapses from 2Ns. These 3Ns ranged from receiving strong net excitation to strong net inhibition, with relatively few neurons near zero (Fig. 4c), consistent with the observation that inputs of the same sign tend to converge (Fig. 4a, b).

We then analyzed the convergence of 2N inputs from different modalities. We hypothesized that 2Ns from modalities of the same valence would tend to provide input of the same sign, whereas modalities of opposing valences may provide input of opposing signs. To address this question, we asked whether net excitation from different modalities onto the same 3Ns is correlated (Supplementary Fig. S1). We found a significant positive correlation of net excitation from sugar and water 2Ns (Supplementary Fig. S1a) and from bitter and IR94e 2Ns (Supplementary Fig. S1f), whereas net excitation for all other pairs of modalities was not correlated. We note that positive correlations may reflect overlap between 2Ns (because 2Ns belonging to multiple modalities were counted as inputs for both modalities) as well as convergent inputs from different 2Ns. Together, these results show that different taste pathways begin to converge at the level of 3Ns, with inputs from the same modality or modalities of the same valence expected to produce net excitation or inhibition.

### 3Ns have feedback connections to 2Ns and GRNs

**Fig. 5:**
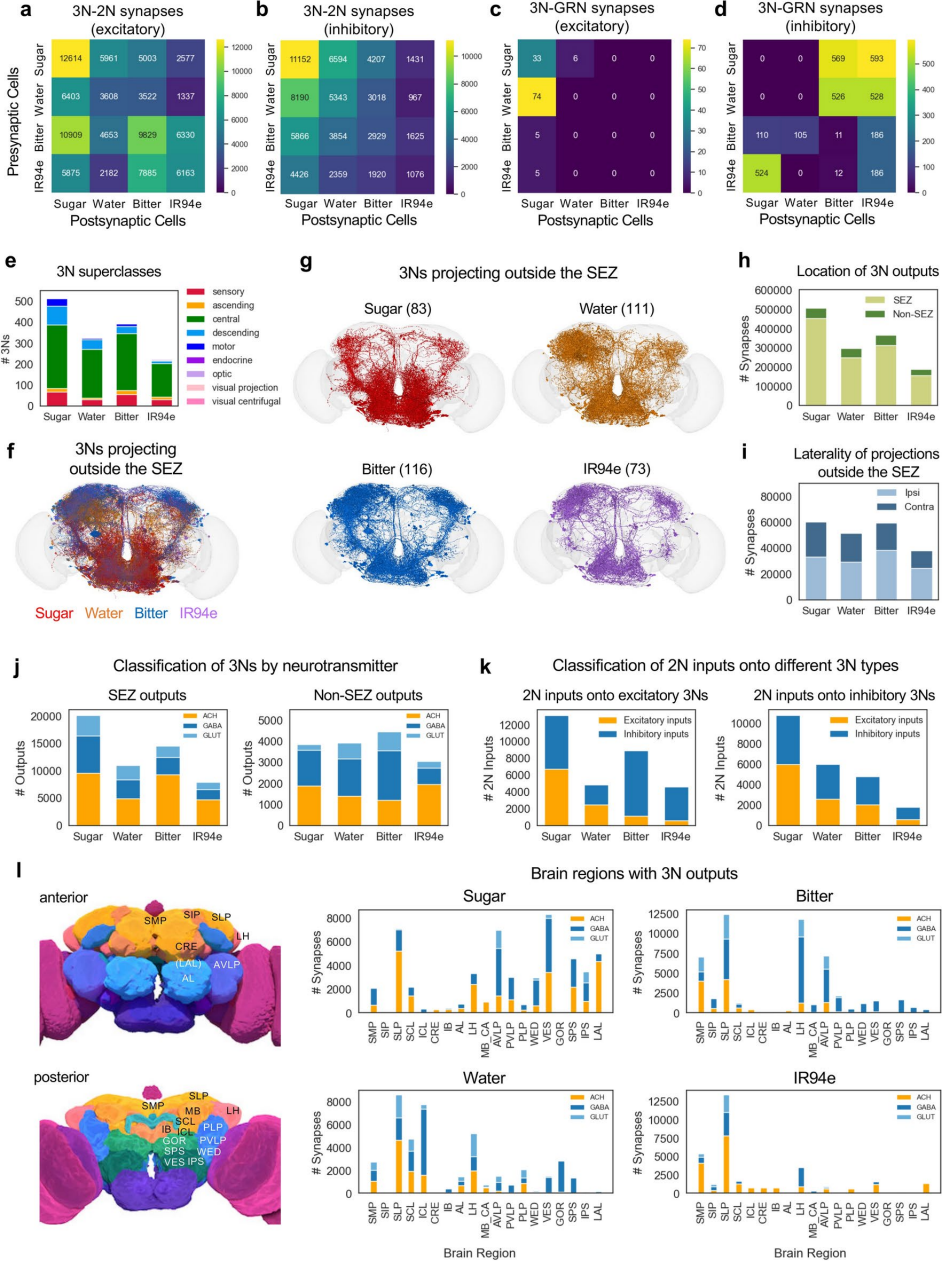
3Ns expand taste processing to additional brain regions. **a**–**d** Heatmaps showing the number of 3N output synapses onto 2Ns (**a**, **b**) or GRNs (**c**, **d**). Excitatory and inhibitory synapses are plotted in separate graphs. **e** Number of 3Ns belonging to each of the 9 superclasses. **f**, **g** Images showing 3Ns of each modality that have output synapses outside of the SEZ. 3Ns for individual modalities (**g**) are shown in addition to the overlaid image (**f**) to facilitate comparisons of projection patterns across modalities, given the density of projections and the substantial overlap between 3Ns of different modalities. Neurons in panel (**f**) are shown at 50% opacity. **h** Number of 3N output synapses that reside within versus outside of the SEZ. All pairwise comparisons of SEZ/non-SEZ proportions between modalities were significantly different (Fisher’s exact test). **i** Number of 3N output synapses outside the SEZ that are located in the ipsilateral versus contralateral hemisphere (relative to the location of GRN projections). All pairwise comparisons of ipsilateral/contralateral proportions between modalities were significantly different except for sugar vs. IR94e (Fisher’s exact test). **j** Predicted neurotransmitters for 3N output connections within the SEZ (left) or outside of the SEZ (right). All pairwise comparisons of the proportion of excitatory vs. inhibitory outputs between modalities were significantly different (Fisher’s exact test). **k** Number of predicted excitatory versus inhibitory 2N inputs onto predicted excitatory (left) or inhibitory (right) 3Ns of each modality. **l** Number of 3N output synapses in brain regions outside of the SEZ, color-coded by neurotransmitter type. The top 12 brain regions for each modality are included, comprising a total of 19 regions that include > 97% of non-SEZ output synapses for sugar, bitter, and IR94e 3Ns and 91% for water 3Ns. Pictures on the left show the approximate location of each brain region (images are from the FlyWire website). Brain regions in parentheses are not visible in the plane shown but are located in a slightly more posterior plane. See text and Methods for abbreviations.

We next classified 3N outputs by their predicted neurotransmitter type and analyzed feedback connections from 3Ns to 2Ns and GRNs (Fig. 5a–d). Both excitatory and inhibitory 3N-2N connections were widespread, with prominent motifs including excitatory and inhibitory connections within the sugar pathway, excitatory connections within and between the bitter and IR94e pathways, excitatory connections from the bitter pathway to the sugar pathway, and inhibitory connections between the sugar and water pathways (Fig. 5a, b). 3N-GRN connections were much sparser and were strongly biased toward inhibition (Fig. 5c, d). The most prominent 3N-GRN connections were inhibitory connections between appetitive and aversive taste modalities, including connections from sugar and water 3Ns to bitter and IR94e GRNs and connections from IR94e 3Ns to sugar GRNs (Fig. 5c, d). These results suggest that excitatory and inhibitory feedback modulates responses early in the taste circuit, with 3N feedback onto GRNs primarily representing cross-modal inhibition.

### 3Ns expand taste processing to additional brain regions

To further characterize the 3Ns, we classified them based on anatomical and functional types. As with the 2Ns, we first identified the superclass of each 3N. The majority of 3Ns were central neurons (58–72%, depending on modality), similar to 2Ns, and 3Ns also included sensory, ascending, descending, motor, and endocrine neurons (Fig. 5e). As compared to 2Ns, a smaller proportion of 3Ns were ascending neurons (2–6%) while, for most modalities, a larger proportion of 3Ns were descending neurons (6–17%). There were some differences across modalities; for example, sugar and water 3Ns included higher proportions of descending neurons (17% and 14%, respectively) than bitter and IR94e 3Ns (9% and 6%, respectively).

We also identified the class of each 3N. Like 2Ns, most 3Ns have not been assigned to a class, but the assigned 3Ns belonged to a wider variety of classes than 2Ns (Table 1). While most 3Ns belonged to the gustatory, ascending, or descending classes, 3N classes also included mechanosensory neurons (for sugar 3Ns), antennal lobe neurons (for sugar and water 3Ns), Kenyon cells of the mushroom body (for water 3Ns), and lateral horn neurons (for bitter and IR94e 3Ns) (Table 1). Because neurons in the antennal lobe, lateral horn, and mushroom body are involved in olfactory processing^29^, some 3Ns are likely to integrate olfactory and taste information.

We then classified 3Ns based on the location of their output synapses in the brain. A small proportion of 3Ns did not have any output synapses in the brain (6% of sugar 3Ns and 1–2% of 3Ns for other modalities), and these neurons were primarily motor or descending neurons. Excluding these neurons, we classified 3Ns based on whether their output synapses reside exclusively within the SEZ (local neurons) or whether some outputs reside outside the SEZ (non-local neurons, which could include neurons projecting out of the SEZ or neurons residing entirely in other brain regions) (Fig. 5f, g). Similar to 2Ns, the majority of 3Ns were local SEZ neurons. However, the proportion of non-local sugar and water 3Ns (17% or 35%, respectively) was substantially higher than the proportion of non-local sugar and water 2Ns (8% or 10%, respectively). Proportions of non-local bitter and IR94e 3Ns were also high (30% or 33%, respectively) but not higher than the proportion of non-local 2Ns for these modalities (31% or 54%, respectively).

Consistent with the preponderance of local 3Ns, a majority of 3N output synapses were located within the SEZ (82–89% for all modalities; Fig. 5h). 3N projections outside of the SEZ were predominantly ipsilateral (relative to the location of GRN projections), similar to 2Ns, but the proportion of contralateral outputs was much higher for 3Ns (35–44%, depending on modality) than for 2Ns (3–34%, depending on modality) (Fig. 5i). Together, these data show that the third layer of the taste pathway involves an expansion to regions beyond the SEZ and into the contralateral hemisphere, but taste processing within the SEZ continues to dominate.

We next analyzed the predicted sign of 3N outputs within and outside of the SEZ. For the sugar and water modalities, 3N outputs within the SEZ were roughly equally distributed between excitatory and inhibitory connections (45–48% excitatory; Fig. 5j, left), similar to sugar and water 2Ns. Interestingly SEZ outputs from bitter and IR94e 3Ns were biased toward excitation (61–64% excitatory; Fig. 5j, left), in contrast to SEZ outputs from bitter and IR94e 2Ns that were biased toward inhibition (see Fig. 2i). Some modalities showed a difference in the proportion of excitatory versus inhibitory 3N outputs when comparing SEZ and non-SEZ synapses (Fig. 5j); for example, bitter 3N outputs within the SEZ were much more likely to be excitatory than those outside of the SEZ. Thus, like 2Ns, the predicted sign of 3N outputs depends on their modality and location.

We then asked whether the sign of taste input onto 3Ns relates to the sign of their output. For example, are inhibitory 3Ns more likely to receive inhibitory input from 2Ns, representing a disinhibitory circuit motif that would cause downstream excitation? To address this question, we analyzed the proportion of excitatory versus inhibitory 2N inputs onto excitatory versus inhibitory 3Ns. For sugar and water 3Ns, the proportion of excitatory 2N inputs was similar for excitatory and inhibitory 3Ns, and many examples of all four possible motifs (excitatory 2N—excitatory 3N, excitatory 2N—inhibitory 3N, inhibitory 2N—excitatory 3N, inhibitory 2N—inhibitory 3N) could be found (Fig. 5k). For bitter and IR94e 3Ns, the proportion of excitatory 2N inputs was higher for inhibitory 3Ns than excitatory 3Ns, and excitatory 3Ns were strongly biased toward receiving inhibitory 2N inputs (Fig. 5k). For these modalities, motifs causing downstream inhibition (excitatory 2N—inhibitory 3N or inhibitory 2N—excitatory 3N) were most common, although motifs for disinhibition (inhibitory 2N—inhibitory 3N) also occurred. Plotting net excitation from 2Ns onto excitatory versus inhibitory 3Ns revealed similar patterns as analyzing the proportion of excitatory and inhibitory inputs (Fig. S2). These results show that GRN-2N-3N circuits can be poised for either activation or inhibition of downstream circuits, with aversive taste modalities showing a bias toward inhibition.

Finally, we analyzed where 3Ns send output projections outside of the SEZ. 3Ns had strong outputs in some of the same areas innervated by 2Ns, including the SMP, SLP, and LH, but 3Ns also projected to additional areas such as the anterior ventrolateral protocerebrum (AVLP) (Fig. 5l). The location of 3N projections varied substantially by modality. For example, sugar 3Ns had many more outputs in the lateral accessory lobe (LAL) and ventromedial neuropils (vest, VES; superior posterior slope, SPS; inferior posterior slope, IPS) than other types of 3Ns. Together, these results show that 3Ns convey taste information both within and outside the SEZ with modality-specific patterns.

### 2Ns and 3Ns receive a variety of inputs from outside the taste system

**Fig. 6:**
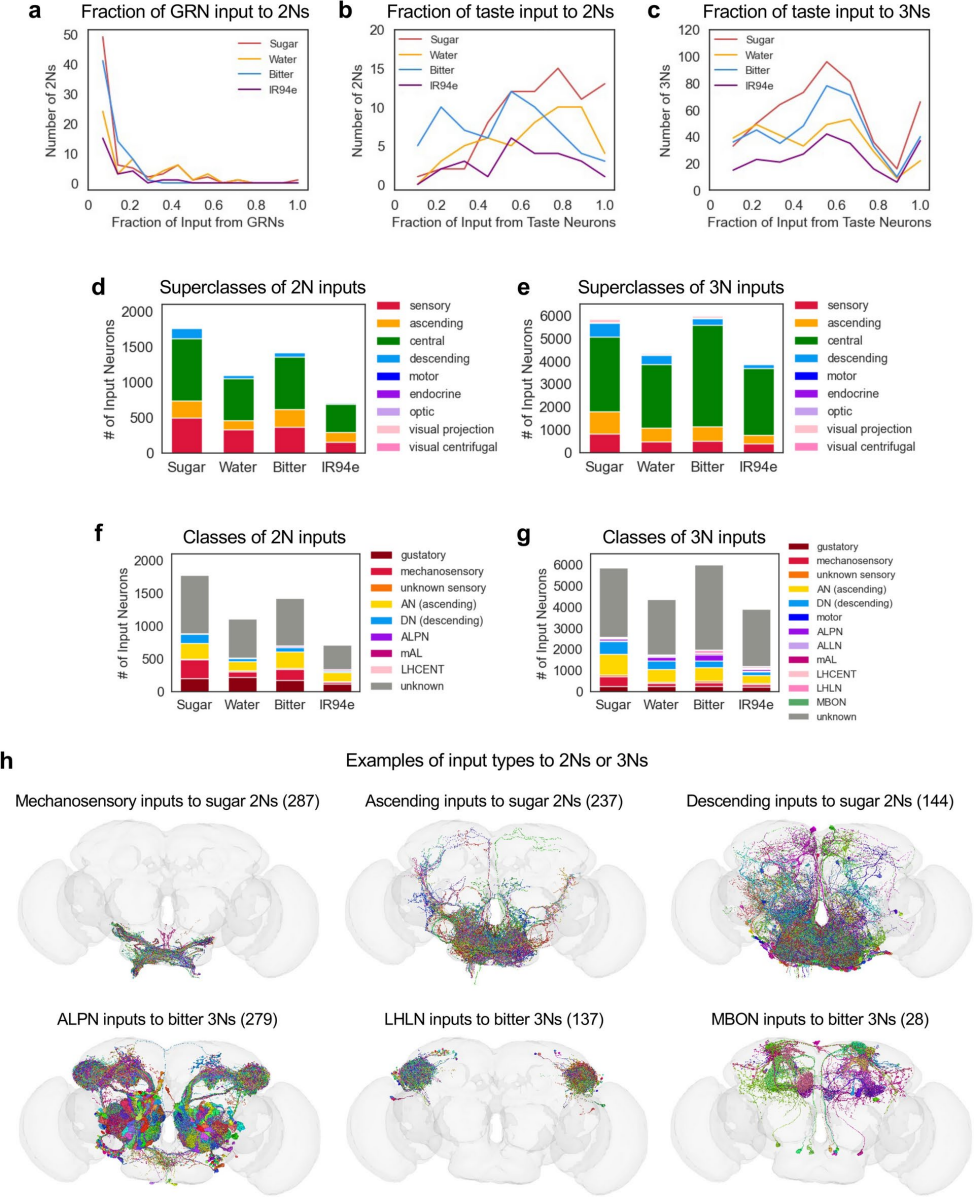
2Ns and 3Ns receive a variety of inputs from outside the taste system. **a** Distribution of the proportion of 2N inputs from GRNs annotated in this study (sugar, water, bitter, or IR94e labellar GRNs on the left side). Median values are 0.10 for water and 0.04 for the other modalities. **b** Distribution of the proportion of 2N inputs from known taste neurons (GRNs, 2Ns, or 3Ns identified in this study). Median values are 0.67 (sugar), 0.63 (water), 0.51 (bitter), and 0.56 (IR94e). **c** Distribution of the proportion of 3N inputs from known taste neurons. Median values are 0.49 (sugar), 0.44 (water), 0.50 (bitter), and 0.52 (IR94e). The peak at 1.0 is primarily due to 3Ns that are sensory or ascending neurons with very few total inputs in the brain. **d**–**e** Number of input neurons to 2Ns (**d**) or 3Ns (**e**) belonging to each of the 9 superclasses. **f**–**g** Classes of input neurons to 2Ns (**f**) or 3Ns (**g**). The top 8 (**f**) or 12 (**g**) classes are shown, which account for > 96% of input neurons for each modality that have been assigned to a class. See text for abbreviations. Note that the “gustatory” class only includes GRNs, not downstream taste neurons. The number of “gustatory” inputs to 3Ns is an underestimate because, by our definition, 3Ns for a given modality cannot receive GRN input from the same modality (otherwise they would be classified as 2Ns). **h** Images showing examples of specific classes of inputs to specific types of 2Ns or 3Ns. Numbers in parentheses denote the number of input neurons in that class, all of which are shown in the images.

We next asked what proportion of input onto 2Ns and 3Ns represents input from the taste system. We found that only a small proportion of inputs onto each 2N (median 4–10%, depending on modality) were from GRNs annotated in this study (sugar, water, bitter, and IR94e labellar GRNs projecting to the left hemisphere) (Fig. 6a). However, the majority of inputs to 2Ns (median 51–67%, depending on modality) were from GRNs, 2Ns, or 3Ns identified in this study, which we refer to as “known taste neurons” (Fig. 6b). Similarly, a large fraction of inputs to 3Ns (median 44–52%, depending on modality) were from known taste neurons (Fig. 6c). Thus, a substantial proportion of inputs to 2Ns and 3Ns are taste-related, and these proportions underestimate the number of taste-related inputs because they do not include labellar input from the right side or input from other taste organs (e.g., leg or pharynx).

To determine what other types of input 2Ns and 3Ns receive, we first identified the superclass of each input neuron. Nearly all inputs to 2Ns and 3Ns belonged to 4 of the 9 superclasses: sensory, ascending, central, or descending (Fig. 6d, e). 2Ns received the most inputs from central neurons (50–56%), followed by sensory (22–30%), ascending (12–19%), and descending (3–8%) neurons (Fig. 6d). 3Ns showed a pattern that was similar but shifted toward central inputs, receiving a majority of inputs from central neurons (56–75%), followed by ascending (9–17%), sensory (8–14%), and descending (5–10%) neurons (Fig. 6e).

We then identified the class of each input neuron to 2Ns or 3Ns. 2N input neurons with an assigned class primarily belonged to four classes: gustatory (i.e., GRNs), mechanosensory, ascending, or descending (Fig. 6f, h). The prominence of mechanosensory inputs was surprising, especially as the number of mechanosensory inputs was comparable to or even greater than the number of gustatory inputs for some modalities (Fig. 6f). 3Ns also received a substantial number of mechanosensory inputs (Fig. 6g).

Aside from gustatory and mechanosensory inputs, 3Ns received inputs from ascending and descending neurons and additional classes including antennal lobe projection neurons (ALPN), antennal lobe local neurons (ALLN), lateral horn centrifugal neurons (LHCENT), lateral horn local neurons (LHLN), mushroom body output neurons (MBON), and mAL neurons (Fig. 6g, h). Inputs from the antennal lobe, mushroom body, and lateral horn suggest that taste and olfactory information is integrated at the 3N layer, as also suggested by our 3N classifications above (Table 1). The classes of inputs to 2Ns or 3Ns of different modalities were largely similar, with some exceptions. For example, bitter 3Ns received more ALPN inputs than 3Ns of other modalities, and bitter and IR94e 3Ns received more LHLN inputs than sugar or water 3Ns (Fig. 6g). Together, these results suggest that early layers of the taste circuit integrate input from a variety of sources, including mechanosensory, olfactory, ascending, and descending inputs.

### Relating connectivity to simulated neuronal activity

A recent study by Shiu et al. developed a computational model to simulate neuronal activity across the fly brain based on each neuron’s connectivity and predicted neurotransmitter^17^. Simulations rely on a leaky integrate-and-fire model, and the parameters of the model (e.g., membrane properties, resting potential, action potential threshold) were fit with experimental data. Although this model has limitations, such as assuming that each neuron has the same biophysical properties, Shiu et al. found that the model was remarkably accurate in predicting activity within sensory-motor circuits, including the sugar taste circuit^17^. We used this model to ask whether the taste 2Ns and 3Ns that we identified are predicted to respond to taste stimulation and what factors determine their predicted response.

**Fig. 7:**
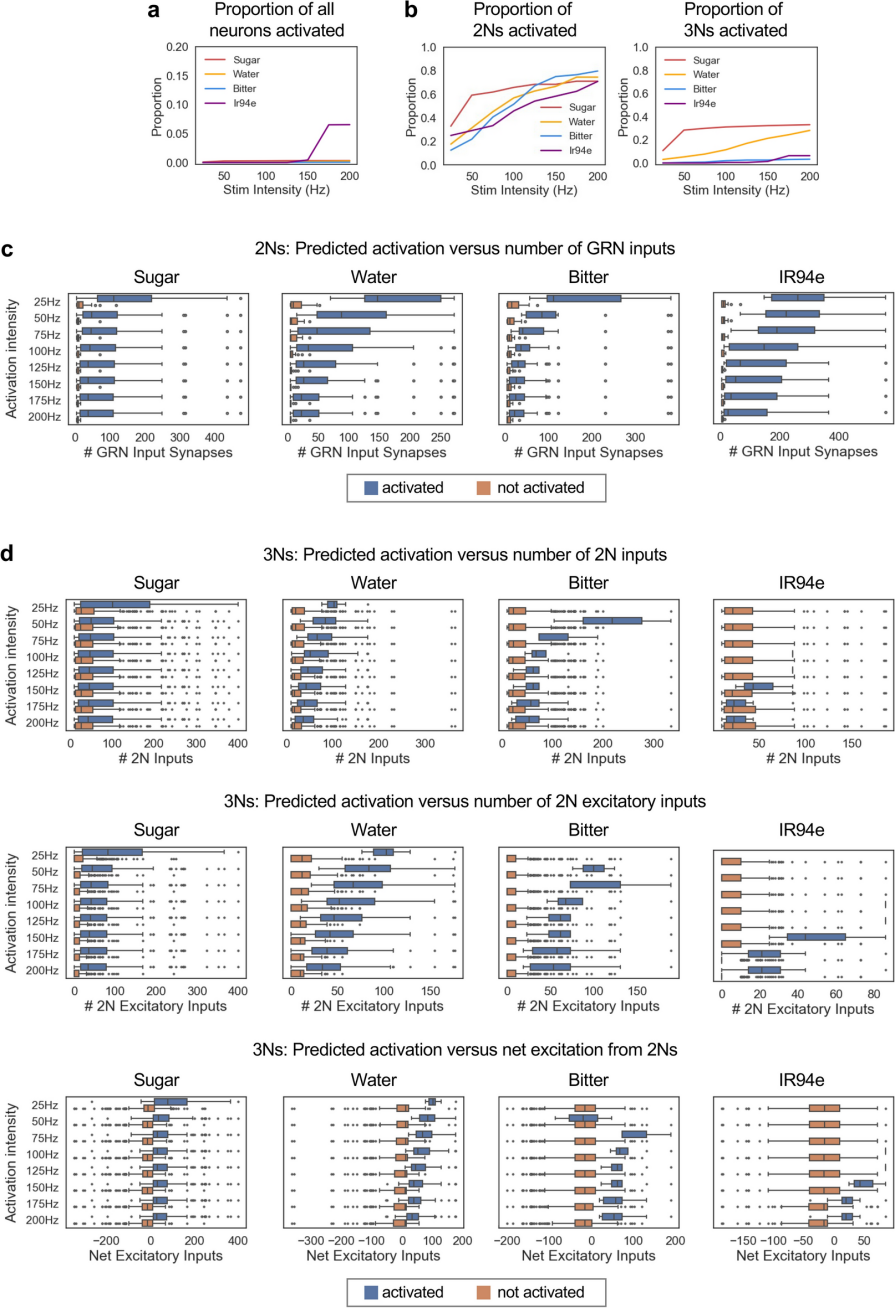
Relating connectivity to simulated neuronal activity. **a** Proportion of all neurons in the brain that were activated by simulated GRN activation at each intensity. Note that the y-axis scale is different from the scale in panel (**b**). Less than 1% of neurons were activated under most conditions, with the exception of IR94e stimulation at the highest intensities, which activated ~ 6% of neurons. **b** Proportion of 2Ns (left) or 3Ns (right) for each modality that were activated by simulated GRN activation at each intensity. **c** Comparison of the number of GRN input synapses for 2Ns that were activated versus not activated in the simulations. **d** Comparison of the number of 2N input synapses (top row), 2N excitatory input synapses (middle row), or net excitatory input synapses (the difference between the number of 2N excitatory and inhibitory input synapses; bottom row) for 3Ns that were activated versus not activated in the simulations. In panels (**c**) and (**d**), boxes represent the interquartile range with a line at the median, bars represent 1.5 times the interquartile range, and points represent outliers outside of this range.

First, we asked how many of the 2Ns that we identified are activated by GRN stimulation in the model. We stimulated GRNs of each taste modality at intensities ranging from 25 to 200 Hz. GRN stimulation generally elicited activity in dozens to hundreds of neurons, corresponding to less than 1% of all neurons in the brain, with the exception of IR94e stimulation at the highest intensities (Fig. 7a). In contrast, the proportion of 2Ns activated by GRN stimulation was 1–2 orders of magnitude higher, ranging from 10 to 60% at lower intensities to 70–80% at higher intensities (Fig. 7b, left). Across all stimulation intensities, 2Ns receiving a larger number of synaptic inputs from GRNs were more likely to be activated (Fig. 7c). The median number of GRN input synapses was typically over 40 for activated 2Ns and less than 10 for non-activated 2Ns, with the disparity being largest at low stimulation intensities. These results suggest that although the majority of 2Ns are expected to be activated by taste input, a substantial proportion of 2Ns may receive GRN input that is too weak to elicit neuronal firing on its own, potentially contributing to neuronal activity in combination with other inputs.

We then asked how many of the 3Ns are activated by GRN stimulation in the model. For each modality, the proportion of activated 3Ns was much lower than the proportion of activated 2Ns, ranging from 0 to 11% at lower stimulation intensities to 3–33% at higher intensities (Fig. 7b, right). A much higher proportion of sugar and water 3Ns were predicted to be activated than bitter and IR94e 3Ns (28–33% for sugar and water versus 3–6% for bitter and IR94e at the highest intensity). Similar to 2Ns, 3Ns receiving a larger number of synaptic inputs from 2Ns were more likely to be activated, but the difference in input between activated and non-activated neurons was not as striking as for 2Ns (Fig. 7d, top). We hypothesized that this may be because 2N inputs to 3Ns can be either excitatory or inhibitory; excitatory 2Ns would promote 3N activation while inhibitory 2Ns would suppress 3N activation. Consistent with this hypothesis, activated versus non-activated 3Ns showed a clearer difference in their number of excitatory 2N inputs (Fig. 7d, middle) or their net excitation from 2Ns (Fig. 7d, bottom) as compared to the number of total 2N inputs (Fig. 7d, top). The median number of excitatory 2N synapses was ~ 40–100 for activated 3Ns and ~ 0–10 for non-activated 3Ns, roughly similar to the numbers for 2Ns. Together, these results show that a lower proportion of 3Ns than 2Ns are predicted to be activated by a given taste input, and this may reflect the use of inhibitory taste coding at the 3N layer as well as the need for excitatory summation from multiple stimuli beyond a single taste input.

## Discussion

In this study, we used a whole-brain connectome to analyze taste pathways in the central brain. We found that 2Ns are largely segregated by taste modality, with the exception of overlap between sugar and water 2Ns. 2Ns primarily convey taste information within the SEZ but also project to a small number of higher-order brain regions in a modality-specific manner. Compared to 2Ns, 3Ns showed more overlap between modalities and more extensive projections outside the SEZ. 3Ns received both excitatory and inhibitory inputs from 2Ns, with input of the same sign tending to converge within a modality. 2Ns and 3Ns received a variety of inputs from outside the taste system, including mechanosensory, olfactory, ascending, and descending inputs. Connectome-based simulations revealed that the majority of 2Ns, but only a minority of 3Ns, are predicted to be activated by GRN stimulation, suggesting that some 2Ns and 3Ns may require additional input for activation or, in the case of 3Ns, may encode taste through an inhibition of activity. Together, these studies provide an overview of early taste processing in *Drosophila* that will guide functional studies in the future.

### Tracing central taste circuits

For decades, central taste circuits in *Drosophila* have remained largely unknown. Initially the field relied on functional or anatomical screens to identify neurons involved in taste processing, revealing interneurons and motor neurons within the SEZ^30–32^. However, pan-neuronal imaging suggested that taste is processed in both the SEZ and the higher brain^33^, consistent with studies showing that taste input is relayed to the mushroom body to mediate taste learning^34,35^. The first systematic approach to identify central taste pathways emerged with the development of trans-Tango^21^, revealing that second-order sugar and bitter neurons comprise large populations that extensively innervate the SEZ with a few projections to the superior protocerebrum^21–23^.

Our analysis of the connectome provides a much more comprehensive and high-resolution view of taste processing at both the second and third layers. Our analyses of 2Ns are largely consistent with results of previous studies, finding that 2Ns reside primarily within the SEZ with selective projections to other brain regions. However, the 2Ns identified from the connectome do not appear to include all of the projection neurons labeled with trans-Tango^21–23^. Some of the missing neurons may receive taste input from neurons outside of the labellum, such as the legs or pharynx, as our study only traced input from labellar GRNs whereas Gal4 drivers for trans-Tango studies labeled GRNs in multiple organs. It is also possible that some neurons identified by trans-Tango represent false positives, as trans-Tango signals are based on proximity whereas connectome data derive from electron microscopy, a much more reliable method to identify synapses.

### Overlap between taste modalities

The extent of overlap between central pathways for processing different taste modalities has been a longstanding subject of debate in both insects and mammals^3,12^. Imaging studies in *Drosophila* suggested that sweet and bitter pathways are largely non-overlapping^33^, but recordings in other insects identified central neurons activated by multiple taste modalities^36,37^. Our analysis of the connectome reveals that 2Ns are largely segregated by modality, with the exception of strong overlap between the two appetitive modalities (sugar and water) and weaker overlap between the two aversive modalities (bitter and IR94e), in addition to weak overlap between water and bitter. Different taste modalities show much stronger convergence at the third layer, with the majority of 3Ns receiving input from multiple modalities.

Our finding that sugar and water pathways show strong convergence aligns with the results of Shiu et al., who used whole-brain simulations to analyze the overlap between neurons activated by each GRN type^17^. A recent imaging study by Li et al. found that taste-responsive cells in the SEZ (which may represent 2Ns, 3Ns, or neurons farther downstream) showed a variety of tuning profiles, with approximately one-third of cells responding to multiple tastes^38^. These findings are consistent with our results showing substantial convergence of inputs from different taste modalities by the third layer. Our analyses revealed more cross-modal convergence (50–76% of 3Ns receive input from multiple modalities) than the proportion of multimodal cells identified by Li et al., but convergence in our study includes both excitatory and inhibitory inputs. Our analyses of lateral and feedback connections reveal that cross-modal inhibition is more common than excitation, and this inhibition would serve to sparsen gustatory tuning. Together, these new studies suggest that the fly taste system contains a diversity of response types, similar to the case in mammals^3^. This diversity of tuning may be important for taste discrimination or for the activation of circuits that mediate different behaviors.

### Lateral and feedback connections

We found that lateral and feedback connections are prominent in early stages of the taste system. Lateral or feedback excitation within a taste modality may create recurrent circuits that generate sustained neural activity^39,40^, which could mediate prolonged behavioral responses or store a memory of the stimulus that modulates future behavior^41^. Conversely, feedback inhibition within a modality would limit stimulus-evoked excitation and could underlie key features of sensory processing such as gain control^42,43^. Inhibitory lateral or feedback connections between different modalities may sparsen the tuning of taste neurons, as observed in other systems^42,44,45^, or may mediate cross-inhibition between pathways driving competing behaviors elicited by different stimuli.

### Brain regions for taste processing

Proboscis motor neurons that control feeding are located in the SEZ^30,46^, suggesting that taste circuits do not need to project out of the SEZ to regulate innate feeding programs. Indeed, a recent study identified a five-layered circuit contained entirely within the SEZ that drives proboscis extension to sugar^25^. SEZ circuits also mediate locomotor stopping in response to sugar through connections to descending pathways^47,48^. However, other behaviors influenced by taste, such as egg-laying^49–51^, associative learning^34,52,53^, or multisensory integration may require taste information to be transmitted to higher brain regions. Our results show that although early taste processing occurs primarily within the SEZ, some neurons relay information to higher brain regions and the extent of non-SEZ processing expands at the third layer. At the second layer, more bitter and IR94e 2Ns project out of the SEZ as compared to sugar and water 2Ns. This difference may reflect specific behavioral programs driven by bitter or IR94e neurons; for example, IR94e 2Ns projecting to the SLP have been implicated in the regulation of egg-laying^18^.

We found that major targets of taste input in the higher brain include the SLP, SMP, and LH. The LH is a well-known olfactory region, as it receives input from second-order olfactory neurons and mediates innate responses to odors^54^. The LH has strong connections to and from the SLP^4^, suggesting that odor and taste inputs may be integrated in these two areas. The SMP contains neurosecretory cells that regulate feeding behaviors, including insulin-producing cells^55^, and SMP neurons regulate sugar and water consumption^56,57^. Interestingly, the projection targets of 2Ns and 3Ns vary substantially based on taste modality. For example, only sugar 2Ns project to the SMP, only water 2Ns project to the mushroom body calyx, and sugar 3Ns have much stronger projections to the LAL and ventromedial neuropils (VES, SPS, IPS) than other 3N types. These differences suggest that certain modalities have more important roles in regulating behaviors linked to those brain regions, such as feeding (SMP) or learning (mushroom body), providing hypotheses for future studies to investigate.

We also observed differences in the balance of excitatory and inhibitory connections based on their anatomical location and modality. Local 2Ns in the bitter and IR94e pathways were more likely to be inhibitory than sugar and water 2Ns, which may reflect the need for bitter and IR94e neurons to inhibit feeding circuits in the SEZ. 2N outputs were more likely to be excitatory if they were located outside of the SEZ than within the SEZ, suggesting that projection neurons relay long-range excitation to circuits that mediate other behaviors. The neurotransmitter composition of 3Ns varied less across modalities than that of 2Ns, but we again observed differences between the excitatory/inhibitory balance of SEZ and non-SEZ outputs that may reflect how taste input is used to regulate feeding circuits in the SEZ compared to circuits for other behaviors outside the SEZ.

### Integration of taste and non-taste inputs

The 2Ns and 3Ns that we identified receive a variety of inputs beyond the taste system. Both 2Ns and 3Ns receive mechanosensory inputs, which likely reflect the need to integrate taste and mechanosensory information when evaluating potential food sources. Flies prefer to feed on soft substrates, and mechanosensory neurons in the proboscis contribute to this preference and regulate motor components of feeding^58–62^. Flies also integrate taste and mechanosensory inputs when choosing an egg-laying substrate^49,50,63,64^.

3Ns receive substantial input from neurons that are likely to process olfactory information, including neurons in the antennal lobe and lateral horn. The integration of smell and taste is likely to be important for food evaluation. It will be interesting to characterize the response properties of multimodal neurons and determine whether these neurons preferentially respond to odors and taste of the same valence.

Both 2Ns and 3Ns also receive a variety of ascending and descending inputs. Ascending neurons may convey input from taste or mechanosensory neurons in the legs^20,65–68^, enabling flies to integrate sensory information from multiple organs. Ascending inputs to the SEZ have also been shown to encode behavioral states and motor actions, such as walking, turning, grooming, and proboscis extension^69^. Sensory circuits may need to integrate information about ongoing behaviors in order to interpret incoming information and determine which potential responses are compatible with current behavior^69^. For example, feeding and walking are mutually exclusive behaviors, and ascending neurons activated during leg movement have been shown to suppress feeding initiation^70^.

Descending neurons convey input to the ventral nerve cord to control actions such as walking^71^, turning^72^, and egg-laying^73^. Many descending neurons have also outputs in the SEZ that help recruit additional descending pathways in order to generate coordinated behaviors^74^. Descending inputs onto 2Ns and 3Ns (some of which are descending neurons themselves) may thus contribute to the coordination of behavioral responses or may transmit information about motor commands (i.e., an efference copy) that modulates how incoming sensory information is interpreted.

### Connecting taste pathways to behavior

This study provides a global view of early taste processing in the fly brain. We did not characterize taste circuits beyond 3Ns because iterative tracing generates larger and larger numbers of neurons at each layer, and within a few steps one would reach a large proportion of neurons in the entire brain^75^. In our whole-brain simulations, we found that stimulation of GRNs activated a large proportion of 2Ns but only a minority of 3Ns. This is partially due to 3Ns that are inhibited rather than excited by taste input, but it is also likely that some 3Ns receive taste input that is too weak to elicit neuronal firing on its own and instead contributes to neuronal firing in combination with other stimuli. Thus, while the number of neurons receiving taste input clearly expands at each layer, the proportion of neurons that receive strong excitatory input may decrease, potentially reflecting a transformation from neurons dedicated to taste processing to neurons with integrative roles.

Recent studies investigating sugar-evoked locomotor stopping and proboscis extension suggest that sensory processing of taste information likely occurs within the first few layers of the circuit, with downstream neurons (fourth-order neurons and beyond) comprising premotor and motor pathways^25,47,48^. Thus, the 2Ns and 3Ns identified here may represent the core neurons that perform sensory processing before downstream circuits implement sensory-motor transformations or higher-order computations. For example, it is notable that 2Ns and 3Ns did not include neurons targeting the central complex, which integrates sensory cues for spatial navigation^76,77^. We expect that integrative brain regions such as the central complex may be targeted by taste neurons downstream of 3Ns.

The taste neurons identified in this study represent candidate neurons whose functional roles can be tested in future work, given the availability of genetic tools to target individual cell types in the SEZ and other regions^6–11^. It will be interesting to compare the taste coding and behavioral roles of neurons belonging to different 2N and 3N populations that we identified, such as excitatory versus inhibitory neurons or neurons projecting to different brain regions, as well as combining neuronal manipulations and recordings to analyze the functional role of lateral and feedback connections. The anatomical insights from this study thus lay the groundwork to understand how the architecture of neural circuits underlies sensory perception and behavior.

## Methods

### Identifying GRNs, 2Ns, and 3Ns

Datasets containing information about neurons, connectivity, and synapse locations were downloaded from FlyWire at https://codex.flywire.ai/api/download. All analyses in this study used version 630. Analyses were performed in Python, and the code and associated datasets needed to perform all analyses in this study are available on GitHub (https://github.com/avdevineni/taste-connectome). Neuron images were generated using the FlyWire web interface.

We focused on tracing the outputs of GRNs in the left hemisphere (referred to in some previous papers^24,25^ as the right hemisphere due to the left–right inversion of the brain volume that was discovered later^4,5^), which have been more thoroughly annotated. GRNs for each modality were identified based on lists from previous studies^17,18^, neuron annotations in FlyWire, and manual inspection of each neuron’s morphology. Our GRN lists are very similar to those used in Shiu et al.^17^ with the following exceptions: we excluded one sugar GRN whose identity as a sugar versus water GRN seemed uncertain, we excluded one bitter GRN that has no connections and is therefore irrelevant for analysis, we excluded 7 IR94e GRNs that have a different morphology from other IR94e GRNs and were not included in IR94e tracing analyses by Guillemin et al.^18^ and we added one sugar GRN that has been annotated as such in FlyWire and appears to have the correct morphology.

We used a connection threshold of 5 synapses to identify 2Ns, which is the threshold suggested by FlyWire to exclude false positives^4^. We set a more stringent threshold of 10 synapses to identify 3Ns (including 10 synapses for both GRN-2N and 2N-3N connections), which reduced the number of 3Ns by ~ 50% compared to a 5-synapse threshold. Analyses of connection stereotypy across hemispheres and brains suggest that a value of ~ 10 synapses represents the threshold for reliable connections^5^. Thus, it is likely that we have identified all reliable 3Ns, whereas some of the 2Ns with weaker GRN input (< 10 synapses) may not be reliably connected to GRNs in all flies. Both 2N and 3N lists excluded GRNs of all modalities; lists of 3Ns excluded neurons that were also 2Ns for the same modality, but not other modalities. The 2N-3N connectivity heatmap (Fig. 3b) uses the same criteria as used for 3N identification, meaning that 2N-2N connections within the same modality are not shown.

### Classifying neurons

We used annotations from the FlyWire v630 dataset to identify the superclass and class of 2Ns, 3Ns, and their input neurons. Note that some of the classes and annotations may have changed in newer FlyWire releases. All classes for 2Ns and 3Ns are shown in Table 1, whereas only the top classes accounting for > 96% of neurons for each modality are shown for 2N and 3N input neurons. Class abbreviations are defined in the text or Table 1 legend, and further descriptions of some classes are described in Schlegel et al.^5^: ALIN includes neurons with dendrites outside and axons inside the antennal lobe, but does not include sensory neurons; ALPN includes neurons with dendrites in the antennal lobe and axonal projections in the protocerebrum, lateral horn or calyx; ALLN includes neurons with both dendrites and axons contained within the antennal lobe; LHCENT includes neurons with dendrites in the protocerebrum and axons in the lateral horn; LHLN includes neurons with both dendrites and axons contained in the lateral horn. mAL neurons were first named in Kimura et al.^78^ as “neurons medially located, just above antennal lobe” and may include sexually dimorphic neurons involved in social behaviors^79,80^ as well as previously characterized taste neurons that do not appear to be dimorphic^23^.

### Analyzing neuronal outputs

Depending on the analysis, we focused on analyzing either output synapses or output connections, with a “connection” comprising all synapses between a single pair of presynaptic and postsynaptic neurons in a given neuropil and requiring a 5-synapse threshold. The term “output” may refer to either an output synapse or output connection, depending on the context, and figure legends specify which metric is being quantified. Analyses of 2N outputs (e.g., analyzing neurotransmitter types or neuropil location) did not exclude outputs to GRNs or other 2Ns; similarly, analyses of 3N outputs did not exclude outputs to GRNs, 2Ns, or other 3Ns. Methods for specific analyses are described further below.

### Analyzing neurotransmitter types and excitatory/inhibitory convergence

For analysis of excitatory and inhibitory neurotransmission, we focused on synapses predicted to use acetylcholine, GABA, or glutamate, as it is not clear whether other predicted neurotransmitters (dopamine and serotonin) would have an excitatory or inhibitory effect on downstream cells. Only a very small fraction of synapses were predicted to use these other neurotransmitters. The FlyWire datasets include neurotransmitter predictions at both the synapse level and the neuron level, with the neuron predictions derived by pooling the synapse predictions and assuming that each neuron only expresses one neurotransmitter according to Dale’s principle^4^. When quantifying the neurotransmitter types for output synapses or output connections, we used synapse-level predictions. When analyzing excitatory/inhibitory convergence onto 3Ns (Fig. 4a, b), we used neuron-level predictions because it was essential to count each 2N-3N pair only once and the synapse-level predictions for the neurotransmitter type of 2N-3N connections sometimes conflicted, even for connections between the same 2N-3N pair. Quantification of net excitation onto 3Ns also used neuron-level predictions.

Analyses of excitatory/inhibitory convergence from 2Ns to 3Ns within an individual modality (Fig. 4a, b) focused on 3Ns receiving exactly two or three 2N inputs, which comprise 86% of all 3Ns receiving more than one 2N input. We excluded cases where at least one input was predicted to use a neurotransmitter other than acetylcholine, GABA, or glutamate. For 3Ns receiving exactly two 2N inputs, our dataset included 71–112 3Ns (112 for sugar, 71 for water, 98 for bitter, and 72 for IR94e), representing 50–97% of all 3Ns receiving more than one 2N input (50% for sugar, 76% for water, 91% for bitter, 97% for IR94e). For 3Ns receiving exactly three 2N inputs, our dataset included 2–53 3Ns (53 for sugar, 12 for water, 8 for bitter, and 2 for IR94e), representing 3–24% of all 3Ns receiving more than one 2N input (24% for sugar, 13% for water, 7% for bitter, 3% for IR94e). Because small sample sizes do not provide reliable data, when presenting results for 3Ns with three 2N inputs we only included data with at least 10 3Ns (sugar and water), and we note that the water data (12 3Ns) should be interpreted cautiously. The expected proportion of 3Ns in each category (all excitatory inputs, all inhibitory inputs, or a combination of excitatory and inhibitory inputs) was calculated using probability rules based on the proportion of excitatory and inhibitory 2N inputs onto the population of 3Ns being analyzed (note that these proportions may differ from those when considering all 2N inputs onto all 3Ns). For example, if the proportions of excitatory and inhibitory 2N inputs are 0.3 and 0.7, respectively, then the expected proportion of 3Ns receiving two excitatory inputs, two inhibitory inputs, or one input of each type is 0.3*0.3, 0.7*0.7, or 2*0.3*0.7, respectively.

### Analyzing projection targets of 2Ns and 3Ns

To classify neuronal outputs within versus outside of the SEZ, we defined the SEZ as encompassing the following regions: gnathal ganglia (GNG), prow (PRW), saddle (SAD), flange (FLA), and cantle (CAN). This definition includes periesophageal neuropils except for the antennal mechanosensory and motor center, which has sometimes been categorized as an SEZ neuropil^9^ but is clearly separated and has specific functions (e.g., auditory processing^81,82^) that are distinct from SEZ processing. To be classified as non-local neurons, 2Ns or 3Ns needed to have at least one output connection outside the SEZ (with a 5-synapse threshold). Classification of laterality for non-SEZ output synapses was based on whether they were located in the left or right hemisphere for brain regions that are lateralized (which includes the majority of brain regions outside the SEZ); we excluded synapses in regions that are not lateralized. When analyzing non-SEZ brain regions receiving 2N or 3N outputs (Figs. 2k and 5l), we pooled outputs located in homologous regions of both hemispheres. Plots of SEZ versus non-SEZ outputs, ipsilateral versus contralateral outputs, and outputs in different brain regions rely on synapse counts, whereas plots categorizing the neurotransmitter types of neuronal outputs rely on the number of output connections.

For plots of brain regions containing 2N or 3N outputs outside the SEZ (Figs. 2k and 5l), we included the regions containing the most numerous output synapses for each modality. For 2Ns, we included the top 6 regions for each modality, comprising a total of 10 regions that included > 96% of non-SEZ output synapses for each modality. For 3Ns, we included the top 12 regions for each modality, comprising a total of 19 regions that included > 97% of non-SEZ output synapses for sugar, bitter, and IR94e 3Ns and 91% for water 3Ns.

Brain region abbreviations not defined in the main text include: superior intermediate protocerebrum (SIP), inferior clamp (ICL), crepine (CRE), inferior bridge (IB), antennal lobe (AL), mushroom body calyx (MB_CA), mushroom body vertical lobe (MB_VL), posterior ventrolateral protocerebrum (PVLP), posterior lateral protocerebrum (PLP), wedge (WED), gorget (GOR).

In the bar graphs, brain regions are grouped by location or function: superior protocerebrum (SMP, SIP, SLP), inferior neuropils (SCL, ICL, CRE, IB), mushroom body (MB_CA, MB_VL), olfactory regions (AL, LH), ventrolateral neuropils (AVLP, PVLP, PLP, WED), ventromedial neuropils (VES, GOR, SPS, IPS), and lateral complex (LAL).

### Whole-brain simulations

Brain simulations were conducted in Python based on code provided publicly by Philip Shiu (https://github.com/philshiu/Drosophila_brain_model), and details about the model are described in Shiu et al.^17^. We used the default parameters for the model, which were chosen by Shiu et al.^17^ based on published experimental data and include: -52 mV resting potential, -52 mV reset potential after a spike, -45 mV spiking threshold, 2.2 ms refractory period, 5 ms tau for synapse decay, 1.8 ms time delay from spike to change in membrane potential, and 0.275 mV synaptic weight. In all simulations, neurons were stimulated for 1 s. All simulations were repeated over 30 trials, and mean values were quantified. We did not apply a threshold for activation; all neurons that had an average firing rate above zero were considered to be “activated” in a given simulation.

### Statistical analyses

Statistical tests are described in the figure legends. Ordinary least squares regression was implemented in Python using the statsmodels module to obtain r-squared values and *p*-values for correlations in Fig. 1g, 3e, and S1. Other statistical tests were performed using GraphPad Prism, Version 9, including Fisher’s exact test to compare proportions between different modalities in Figs. 2g–j and 5h–j, and chi-squared test to compared observed versus expected values in Fig. 4a, b.

## Supplementary Information

Supplementary Information.

## Data Availability

The code and associated datasets needed to perform all analyses in this study are publicly available on GitHub at https://github.com/avdevineni/taste-connectome, and all data reported in this paper can be obtained using this code. Data files containing lists of second- and third-order neurons identified in this study (using the code described above) are also available in the same GitHub repository, and any additional data files will be shared upon request to the corresponding author.
