# Supplementary material for: Connectomic analysis of taste circuits in Drosophila
Source: Sci Rep. 2025 Feb 12;15:5278. doi: 10.1038/s41598-025-89088-9 (PMC11821855; doi:10.1038/s41598-025-89088-9)
Supplement: Supplementary file 1 — Supplementary Information. [file 41598_2025_89088_MOESM1_ESM.pdf]

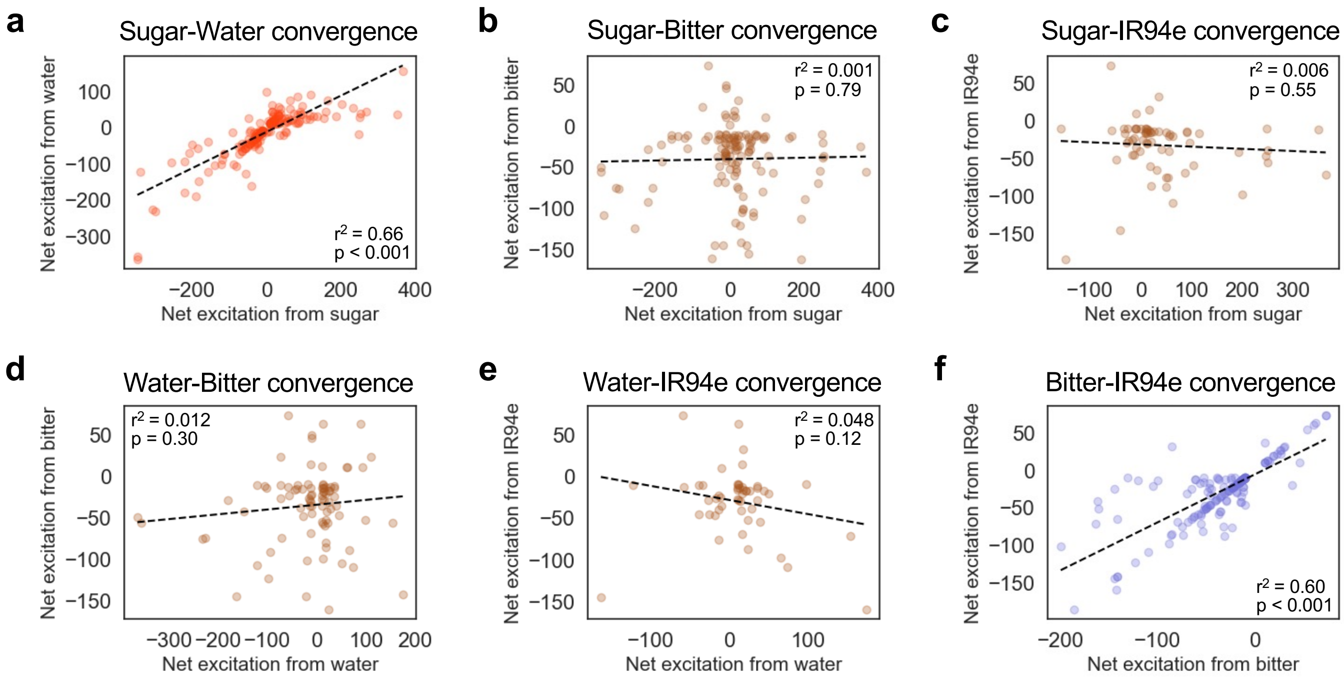

Supplementary Figure S1. Net excitation from 2N inputs of different modalities onto common 3Ns

3Ns receiving input from multiple modalities were analyzed to determine whether net excitation from 2Ns of different modalities is correlated. Plots are color-coded to show whether convergence between two appetitive modalities (red-orange, panel a), two aversive modalities (blue-purple, panel f), or a mix of appetitive and aversive modalities (brown) is being analyzed. Significant correlations were only observed when the input modalities had the same valence (sugar and water, panel a; bitter and IR94e, panel f). Note that 2Ns belonging to multiple modalities (see Figure 1e) were counted as inputs for both modalities, which could contribute to positive correlations (and accounts for data points along the line of equality).

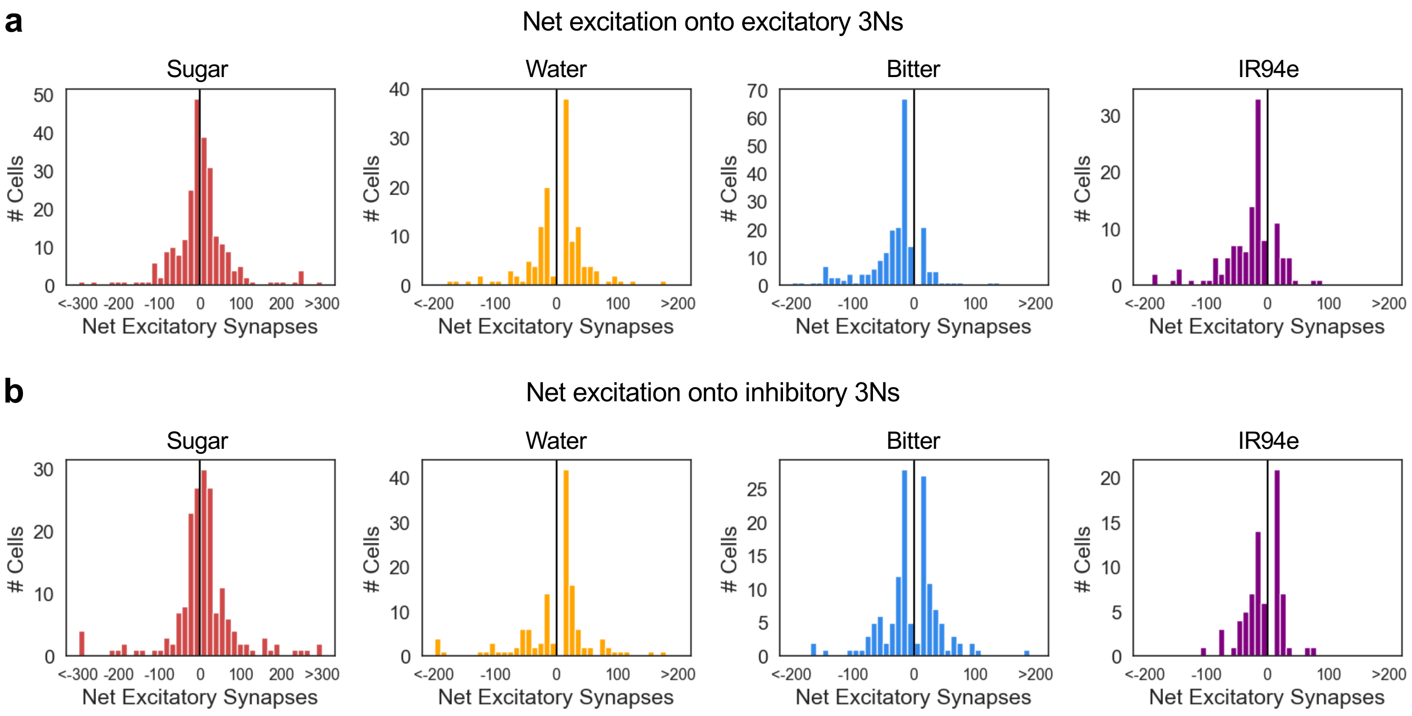

Supplementary Figure S2. 2N inputs onto excitatory versus inhibitory 3Ns

Distribution of net excitation onto each excitatory (a) or inhibitory (b) 3N for each modality. Net excitation represents the difference between the number of excitatory and inhibitory 2N input synapses; positive numbers represent net excitation and negative numbers represent net inhibition. The distribution of net excitation is skewed toward inhibition for excitatory bitter and IR94e 3Ns, similar to the data shown in Figure 5k.
